# Supplementary material for: Integrating Molecular Diagnostics and GIS Mapping: A Multidisciplinary Approach to Understanding Tuberculosis Disease Dynamics in South Africa Using Xpert MTB/RIF
Source: Diagnostics (Basel). 2023 Oct 10;13(20):3163. doi: 10.3390/diagnostics13203163 (PMC10606157; doi:10.3390/diagnostics13203163)
Supplement: Supplementary file 1 [file diagnostics-13-03163-s001.zip › diagnostics-2562240-supplementary.pdf]

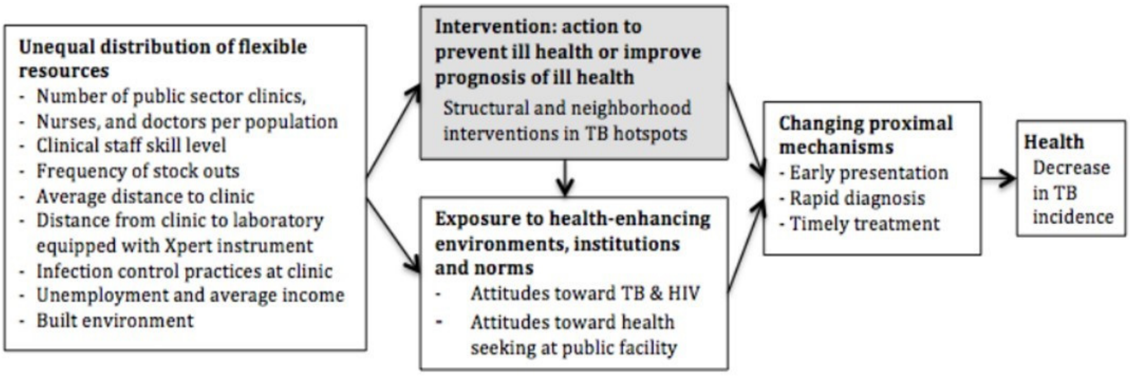

Figure S1. Conceptual framework of the fundamental causes of TB transmission.

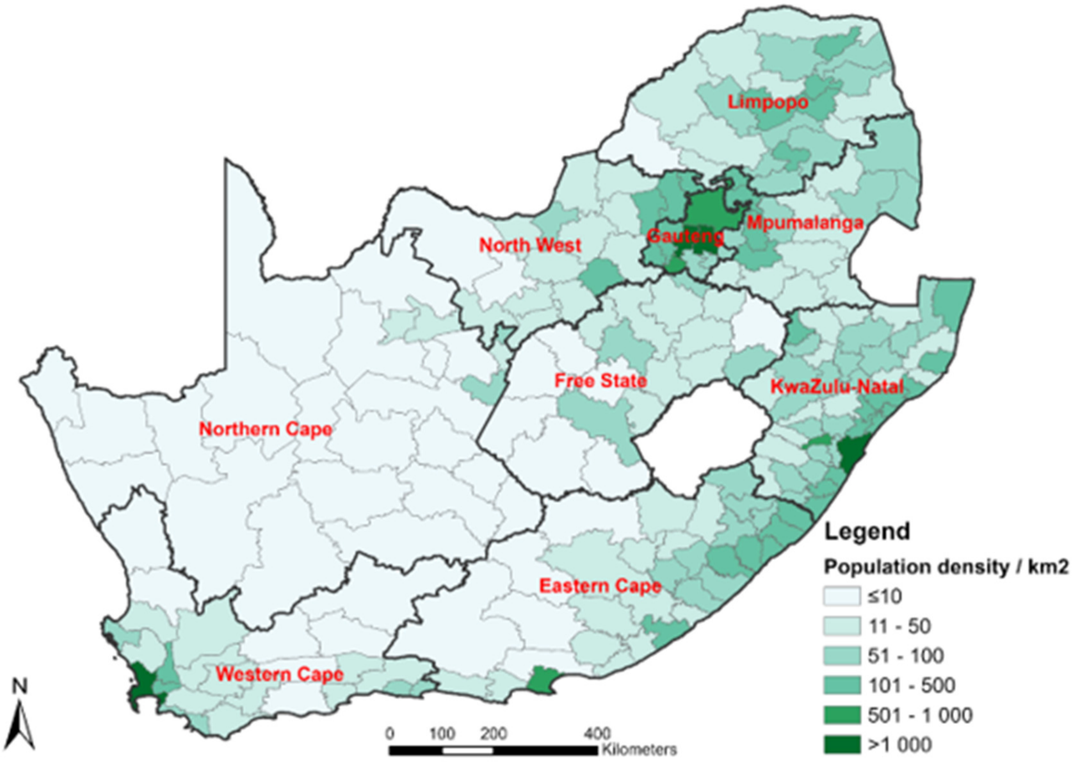

.

Figure S2. South African provinces and population density.

Table S1. Univariate logistic regression analysis of risk factors associated with high mycobacterial load in the Eastern Cape (2013–2016).

Variable	OR	95% CI	p-value
Median patient Age	0.86	0.79 - 0.93	<0.001
Population Count	1.00	1.00 - 1.00	0.002
Number of TB patients in 2015	1.06	1.02 - 1.10	0.004
Number of non-specified health care workers	0.76	0.62 - 0.93	0.007
Proportion of initial default among those positive for TB	1.93	1.19 - 3.13	0.008
Number of health care workers that are nurses	1.57	1.05 - 2.37	0.03
Land use (urban)	49.64	5.92 - 416.08	<0.001
Number of MDR-TB patients diagnosed at clinic in 2015	2.19	1.00 - 4.81	0.051
Approximate number of patients per day	1.01	0.99 - 1.03	0.191
Does your facility have a TB infection control (IC) officer? (yes)	2.10	0.67 - 6.61	0.204
How much does it cost patients to travel to the facility?	0.98	0.95 - 1.02	0.33
Does your facility have DOTS supporters?(yes)	0.43	0.08 - 2.43	0.341
Treatment success rate in 2015	0.94	0.75 - 1.17	0.567
Frequency of laboratory courier per day	0.93	0.74 - 1.18	0.567
How often is TB screening performed?	0.93	0.70 - 1.23	0.601
How far do patients travel to the facility?	0.93	0.69 - 1.25	0.625
Number of DOTS supporters	0.95	0.74 - 1.21	0.653
Number of health care workers that are doctors	1.22	0.45 - 3.29	0.697
Is annual surveillance of HCW conducted?	1.22	0.44 - 3.40	0.703
Turnaround time from sputum to result	1.01	0.96 - 1.05	0.756
How long does it take patients to reach the facility?	1.00	0.99 - 1.01	0.829
Type of facility (PHC)	1.20	0.16 - 9.14	0.86
Turnaround time from sputum collection to treatment initiation (TB positives)	1.00	0.98 - 1.02	0.997
